# Machine Learning Nucleation Collective Variables with Graph Neural Networks

**DOI:** 10.1021/acs.jctc.3c00722

**Published:** 2023-10-25

**Authors:** Florian M. Dietrich, Xavier R. Advincula, Gianpaolo Gobbo, Michael A. Bellucci, Matteo Salvalaglio

**Affiliations:** † Thomas Young Centre and Department of Chemical Engineering, University College London, London WC1E 7JE, U.K.; ‡ XtalPi Inc., 245 Main Street, Cambridge, Massachusetts 02142, United States

## Abstract

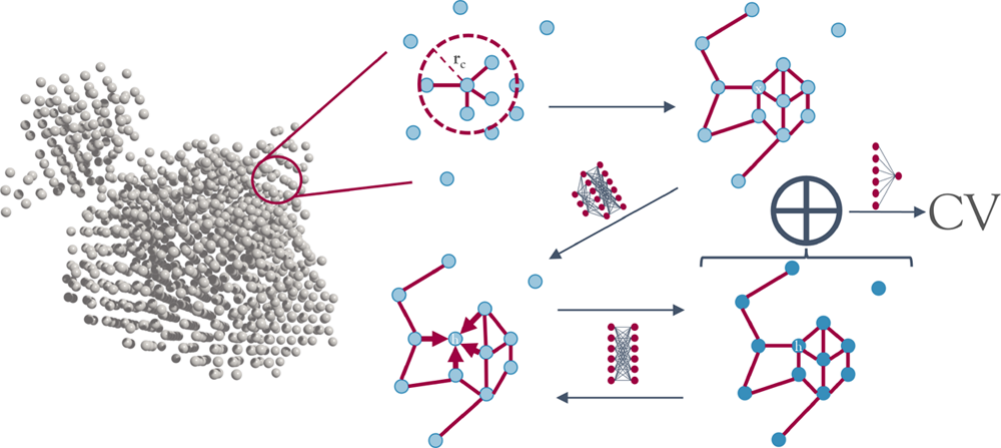

The efficient calculation of nucleation collective variables (CVs) is one of the main limitations to the application of enhanced sampling methods to the investigation of nucleation processes in realistic environments. Here we discuss the development of a graph-based model for the approximation of nucleation CVs that enables orders-of-magnitude gains in computational efficiency in the on-the-fly evaluation of nucleation CVs. By performing simulations on a nucleating colloidal system mimicking a multistep nucleation process from solution, we assess the model’s efficiency in both postprocessing and on-the-fly biasing of nucleation trajectories with pulling, umbrella sampling, and metadynamics simulations. Moreover, we probe and discuss the transferability of graph-based models of nucleation CVs across systems using the model of a CV based on sixth-order Steinhardt parameters trained on a colloidal system to drive the nucleation of crystalline copper from its melt. Our approach is general and potentially transferable to more complex systems as well as to different CVs.

## Introduction

Nucleation, the formation of the first stable embryo of a new phase from an out-of-equilibrium mother phase, lies at the heart of material synthesis in both nature and industry. Nucleation, in fact, controls the structural characteristics of the product material and determines the kinetics of the formation of different polymorphs of the same compound.^1−4^ As such, modeling nucleation to predict its characteristic rate and to understand its molecular mechanisms in realistic conditions remains one of the grand challenges in the field of molecular modeling and simulation.^5−9^ Moreover, under the effect of realistic thermodynamic driving forces, nucleation is a paradigmatic example of a rare event occurring over time scales that far exceed those that can be accessed by brute-force molecular dynamics simulations.^7−10^ Consequently, molecular simulations aimed at probing nucleation mechanisms at the atomic scale are unfeasible with brute-force simulations and require the use of enhanced sampling methods and more complex collective variables (CVs).

Enhanced sampling simulations often require tracking the reaction progress along a low-dimensional set of CVs that, ideally, approximate the reaction coordinate^11^ associated with the nucleation process. On-the-fly calculation of CVs is a requirement of most *unseeded* enhanced sampling simulation methods aimed at studying nucleation, where the formation of a stable nucleus is modeled starting from a homogeneous and supersaturated solution rather than a supersaturated solution seeded with crystal nuclei as is commonly done in *seeded* methods. The unseeded methods include *biased* enhanced sampling methods as well as path sampling methods such as forward flux sampling, which relies on the calculation of CVs to track the progress of a rare transition.^12−14^ Biased enhanced sampling methods utilize biasing potentials, or forces, which are functions of the CVs that are added to the system’s Hamiltonian to facilitate the exploration of configuration space. Such methods, including umbrella sampling,^15^ metadynamics,^16,17^ adaptive biasing force,^18^ and adiabatic bias molecular dynamics^19^ with all their variants,^20^ require the calculation of CVs and their gradient to propagate the biased dynamics and accelerate the frequency of rare events.

CVs are functions of the microscopic coordinates of the system capable of distinguishing the relevant macrostates involved in the activated transformation, and thus, they approximate the reaction coordinate associated with the transformation. In contrast with other domains, such as conformational dynamics, ligand binding, and protein folding, where the computational overheads associated with the calculation of CVs are usually minimal, in simulations of nucleation, effective CVs are inherently more complex and computationally expensive. The computational cost associated with nucleation CVs is related to the fact that, regardless of the underlying principles used to mathematically formulate nucleation CVs, the process of assembly emerges from the collective evolution of all the growth units (atoms, particles, or molecules) simulated. As a consequence, CVs are typically formulated as combinations of descriptors of the local atomic environment of the growth units.

To be physically meaningful, such combinations are required to be invariant to the roto-translations of the system as well as invariant to the permutation of chemically equivalent growth units.^7,21,22^ Hence, most CVs are constructed by combining roto-translationally invariant local symmetry functions via permutationally invariant operators that are exclusively a function of the system coordinates. A characteristic reference model for the mathematical structure of such CVs is portrayed by the Steinhardt order parameters,^23^ a prototypical example of nucleation CVs. In Steinhardt order parameters, rotationally invariant spherical harmonics are computed on the basis of a set of distances between growth units representing a local environment, and in their original formulation, functions of the spherical harmonics are combined in a permutationally invariant average, yielding a CV able to describe the state of a system undergoing crystal nucleation.

Starting from the Steinhardt order parameters, many different CVs for the study of nucleation and, more generally, of crystallization have been proposed,^7,22^ significantly expanding the scope and ability to describe crystalline systems from atomic lattices to more complex molecular assemblies.^21,24−27^ Despite the differences in the mathematical principles leveraged to characterize the local environments, the structure by which invariances to roto-translation and permutation are achieved is common to the vast majority of nucleation CVs. Similar to the problem of characterizing local environments via symmetry functions to train machine learning potentials (MLPs), the computational bottleneck in the efficient computation of nucleation CVs is the scalable calculation of the local symmetry functions^28^ needed to ensure roto-translational invariance.

In this work, we have developed a graph-based model able to efficiently approximate nucleation CVs, bypassing the need for an on-the-fly computation of symmetry functions and their gradient in favor of a much more efficient evaluation of a function of the molecular graph constructed from the atomic coordinates. We show that this approach allows for an efficient size and system transferability and enables order-of-magnitude efficiency gains compared to those of the direct evaluation of classical CVs. It should be noted that our aim in this work is to provide a computationally efficient alternative to the direct calculation of nucleation CVs. The development, validation, and optimization of CVs for the study of nucleation processes, which remains a very active area of research, is not part of the aim of this work. Nevertheless, we note that many approaches focused on the identification of better CVs for the description of complex transitions are based on the combination of large numbers of order parameters. This applies to both data-driven assessments^29,30^ and optimization strategies^31^ of nucleation reaction coordinates, which would greatly benefit from the deployment of faster models able to approximate expensive CVs, thus enabling studies involving a larger (and more physically significant) number of growth units.

The model architecture and several functionalities to facilitate the convenient construction of these approximations are implemented in the NNucleate Python library, developed to accompany this work. This library can be installed from https://github.com/mme-ucl/NNucleate, and its documentation is hosted under https://flofega.github.io/NNucleate/html/index.html.

## Theory

**Figure 1:**
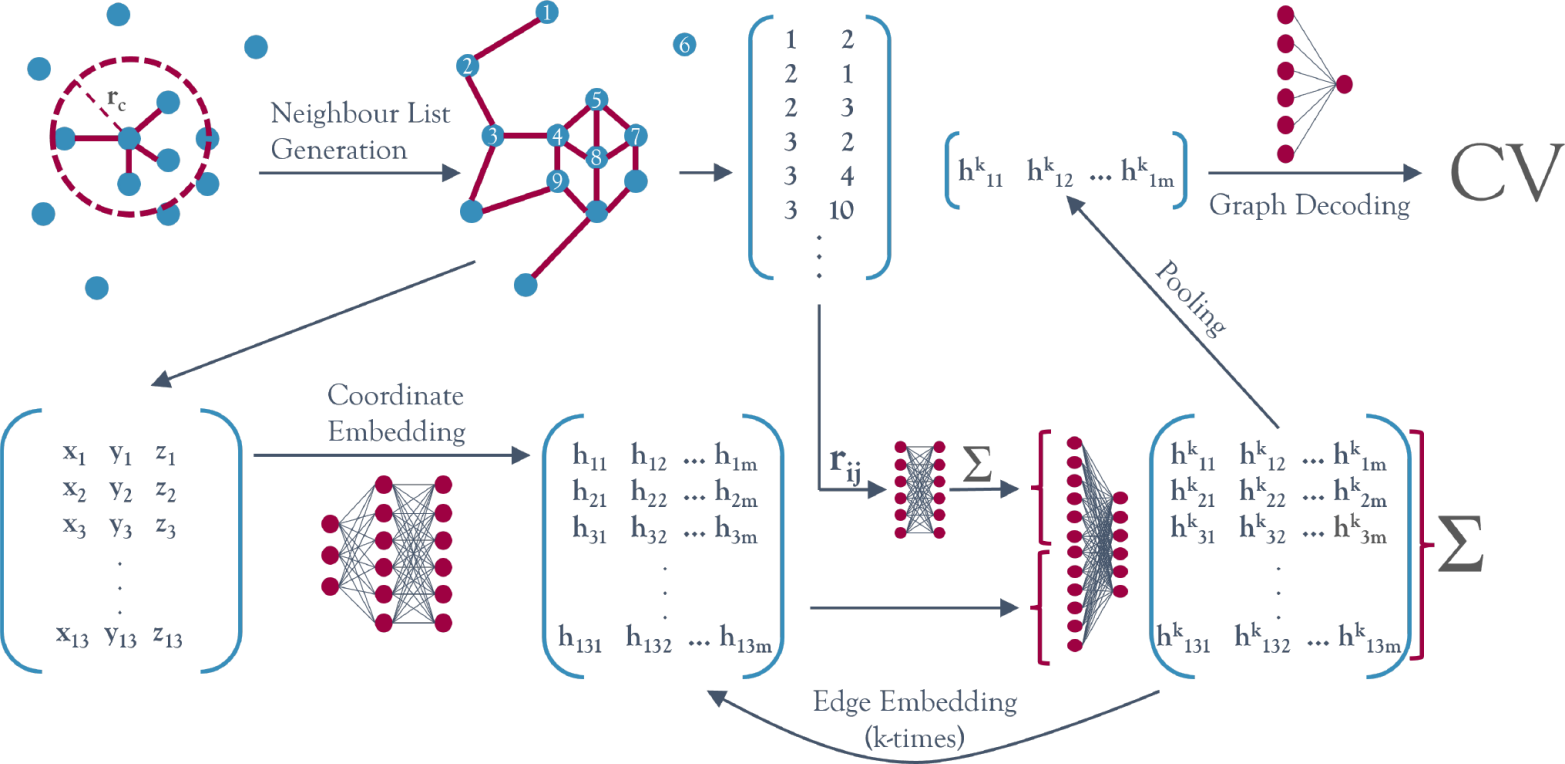
A GNN model for learning nucleation CVs. This figure shows a schematic depiction of the method developed. In the first step, the molecular/atomic graph is constructed using a neighbor list algorithm. The Cartesian coordinates contained in each node are embedded into a higher-dimensional representation via the row-wise application of a multilayer perceptron. Then each row is repeatedly updated with its neighborhood information as indicated by the edges of the graph. After the user-defined amount of edge embeddings, all the local predictions are pooled via summation and mapped from the *m*-dimensional internal representation to a one-dimensional final prediction.

The graph-based architecture to approximate nucleation CVs in atomistic and molecular systems is graphically summarized in Figure 1. A graph neural network (GNN) is a function acting upon a graph, as defined by a set of nodes and edges . Since such a graph is defined only by the relative relations between nodes, it is invariant to any edge-preserving permutation of the nodes. Furthermore, any function acting on the graph would inherit this invariance. This property makes a GNN desirable for the calculation of nucleation CVs, as any approximator for any global descriptor of an atomistic system must be invariant to the permutation of chemically equivalent particles.

Here, each node **x**_*i*_ contains the set of Cartesian coordinates describing particle *i*. In general, a function *f* evaluating properties of the set of nodes **X** makes a graph-level CV prediction by pooling node-level predictions in a permutationally invariant manner:
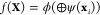
1where ⊕ is a permutationally invariant pooling function (e.g., sum or max), ψ is a node-level predictor, and ϕ is a graph-level predictor, where predictor refers to a learnable mathematical function that maps the node inputs to node-level predictions and the pooled node-level predictions to a global prediction, respectively. The latter function is implemented as a multilayer perceptron and referred to in Figure 1 as the graph decoder.

To consider the connectivity of the graph, when making predictions, the node-level predictor ψ should depend on the central node as well as its edges in the so-called edge-embedding step. However, a function making such a prediction has to again be permutationally invariant to the order in which the neighboring nodes are considered. This is achieved again by pooling the predictions on individual neighbors:
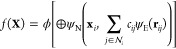
2Here the indices N and E differentiate between node and edge-level predictions, respectively. ψ_N_ is implemented as a multilayer perceptron that maps the vector resulting from stacking the node vector and the edge contribution back to the dimensionality of the node vector. ψ_E_ is another multilayer perceptron that acts on the relative distance vector **r**_*ij*_ = **x**_*j*_ – **x**_*i*_, mapping it to an edge-level prediction of the same dimensionality. When making these design choices together with constant edge weights *c*_*ij*_, the resulting function is often referred to as a graph-convolutional layer.^32,33^ is the neighborhood of node *i* as defined by its undirected edges:
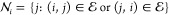
3In this work, the edge weights are assumed to be constant and set to 1. The reason is that, on the one hand, the computational cost of this approach should be minimized for the application in mind. On the other hand, this step is justified by the fact that a strong bias about the importance of a particular node is already introduced through the way the graph is constructed.

With this approach, any graph-level prediction is based on node-level predictions, which consider the nodes in their immediate neighborhood. However, this framework allows for the inclusion of longer-range information by repeating the node-embedding step multiple times, where after the first edge embedding (see Figure 1) the internal representation **h**_*i*_^0^ contains information about its immediately adjacent nodes. Repeating this process *k* times will embed information from nodes that are *k* edges removed from node *i*, resulting in the internal representation **h**_*i*_^*k*^:
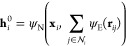
4
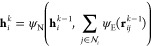
5
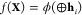
6

This approach, in many ways, mirrors the mathematical structure of the analytical CV. The model predicts local contributions based on pairwise neighbor interactions and pools them into a permutationally invariant final prediction. This structural similarity can be further highlighted by rewriting the equation for the CV *n*(*Q*6) (see Methods) with a style and notation mirroring that of eq 2, as indicated by OP(**X**), representing a general order parameter as a function of the coordinates of the system **X**:
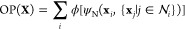
7where
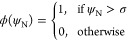
8and
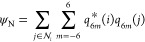
9where σ is a threshold for a characteristic degree of local order and *q*_6*m*_(*i*) is the sixth-order Steinhardt parameter as defined in Methods.

A key difference, however, is that the predictions of this model are not E(3)-invariant. The model has to learn from data how rotations and translations at the local level affect the value of the global CV based on Cartesian coordinates. This task is rendered more tractable by working in reduced coordinate space and evaluating the edge-level prediction ψ_E_ over the relative distance vector **r**_*ij*_ = **x**_*j*_ – **x**_*i*_ instead of the absolute position of the neighbor. This step is key for the objectives of the model, as it allows us to bypass the calculation of expensive rotationally invariant descriptors of the local environment, thus enabling significant gains in computational efficiency over the direct calculation of CVs.

In fact, the rate-limiting step in the evaluation of the model is the construction of the graph. There are different ways one could construct the atomic graph, but the most efficient, under the consideration of periodic boundary conditions, is to construct the neighbor lists for each particle. Another advantage of the neighbor list graph is that the resulting hyperparameter, i.e., the cutoff radius, can be interpreted as the radius of the first coordination shell. Thus, it is set to the position of the first minimum of the radial distribution function of the respective system throughout this work.

One last problem with the model proposed in eq 2 is that the node-level predictions are inherently subject to numerical noise. This noise averages globally, allowing for accurate global predictions. However, when the model is deployed in biased simulations, issues may appear due to this noise. In fact, when biasing with an analytical CV, the force is only applied to a set of relevant atoms that have a non-null contribution to the CV, whereas in the model CV, no force component is exactly zero, even when particles have a null coordination number. This can negatively affect the system’s dynamics when biasing with high force constants or crossing high energy barriers with metadynamics. This issue is remedied by setting the gradient components corresponding to particles with no neighbors to zero before passing them to the enhanced sampling code.

## Methods

All molecular dynamics simulations in this work are performed in the canonical ensemble, tempered to 2T* with 421 particles in a cubic box of length 92.83σ using LAMMPS.^34^ The colloidal particles are modeled via a Derjaguin–Landau–Verwey–Overbeek (DLVO) potential with a cutoff of 12.5σ. Details of the potential, its expected thermodynamic behavior, and additional simulation details are available in ref (30).

The CVs used to describe the nucleation mechanisms of the colloidal system of interest are *n* and *n*(*Q*6). The variable *n* describes the number of particles with a coordination number *c*_*i*_ larger than a threshold and thus counts the number of particles in the dense liquid droplet that forms in the lead-up to a nucleation event.^35^ The coordination number of particle *i*, *c*_*i*_, is computed as
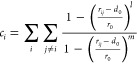
10where *r*_*ij*_ = |**r**_*ij*_| is the distance between particle *i* and its *j*th neighbor. The parameters *l*, *m*, *d*_0_ and *r*_0_ determine the shape of the switching function. Their exact values can be found in ref (30) and in the PLUMED input files on PLUMED-NEST (https://www.plumed-nest.org/, plumID:23.026).

The variable used to quantify the size of crystalline domains in the system, *n*(*Q*6), counts the number of particles characterized by a local Steinhardt parameter value *Q*6_*i*_ above a set threshold.^23,36−38^ The order parameter *Q*6_*i*_ is defined as
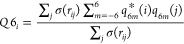
11where σ(*r*_*ij*_) is a switching function acting on the pairwise distance *r*_*ij*_ and is equal to 1 inside the radius of the first coordination shell of the central particle and smoothly decays to 0 at 8 reduced distance units. The shape of the switching function is implemented in PLUMED under the RATIONAL keyword and is structurally identical to eq 10. *q*_6*m*_(*i*) is the sixth-order Steinhardt parameter of particle *i*, defined as
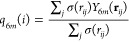
12where *Y*_6*m*_(**r**_*ij*_) is the *m*th component of a sixth-order spherical harmonic. The complex norm *q*_6m_^*^(*i*)*q*_6*m*_(*j*) gives a measure of how much the orientation of the coordination shell of particle *j* matches that of the central particle *i*.

All machine learning models in this work were trained by using the NNucleate package. This package is built on top of PyTorch.^39^ It utilizes functionalities from the MDTraj^40^ and MDAnalysis^41,42^ packages to train CVs, augment and manage datasets, analyze models, and translate models into PLUMED-readable CV files. These Python scripts are used as CVs through the PLUMED2 fork PyCV.^43^ Converting the models into a format supported by PyCV involves Alphabet’s Jax and Flax packages, and the necessary gradients are obtained using Jax’s autodifferentiation implementation.^44,45^

The model optimizations were performed using the mean-square error metric, Adam optimizer, and typically a learning rate of 1 × 10^–3^.^46^ The main hyperparameters of the model, the number of latent dimensions and the number of graph convolutional layers, were optimized using the asynchronous hyperband (ASHA) procedure as implemented in Ray Tune.^47,48^

## Results and Discussion

### Is a GNN Model *Really* Necessary?

The first thing to consider when developing an approximation for a nucleation CV is that the final approximation needs to respect the same invariances as the underlying CV. Namely, the value of our approximation needs to be invariant to the identity of the particles in the cluster, the orientation of the cluster, and the absolute position of the cluster in space. The most naive and potentially most efficient solution to this problem is to start with a multilayer perceptron that maps the Cartesian coordinates of the system directly to an approximated CV value. The assumption then would be that the model can learn all of these invariances via “brute force” by just observing enough training data. In a sense, that would shift the computational cost from production to a training process that has to be performed only once. Such neural networks are, in theory, sufficiently powerful for this task and inexpensive to evaluate. However, this type of neural network is not size-transferable or applicable outside of its training domain. Therefore, a more apt formulation of the training goal, rather than learning the CV, would be learning to resolve all degeneracies of the CV in Cartesian space within a given training domain. This requires the model to learn the inherent invariances of the CV from a sufficiently large dataset.

**Figure 2:**
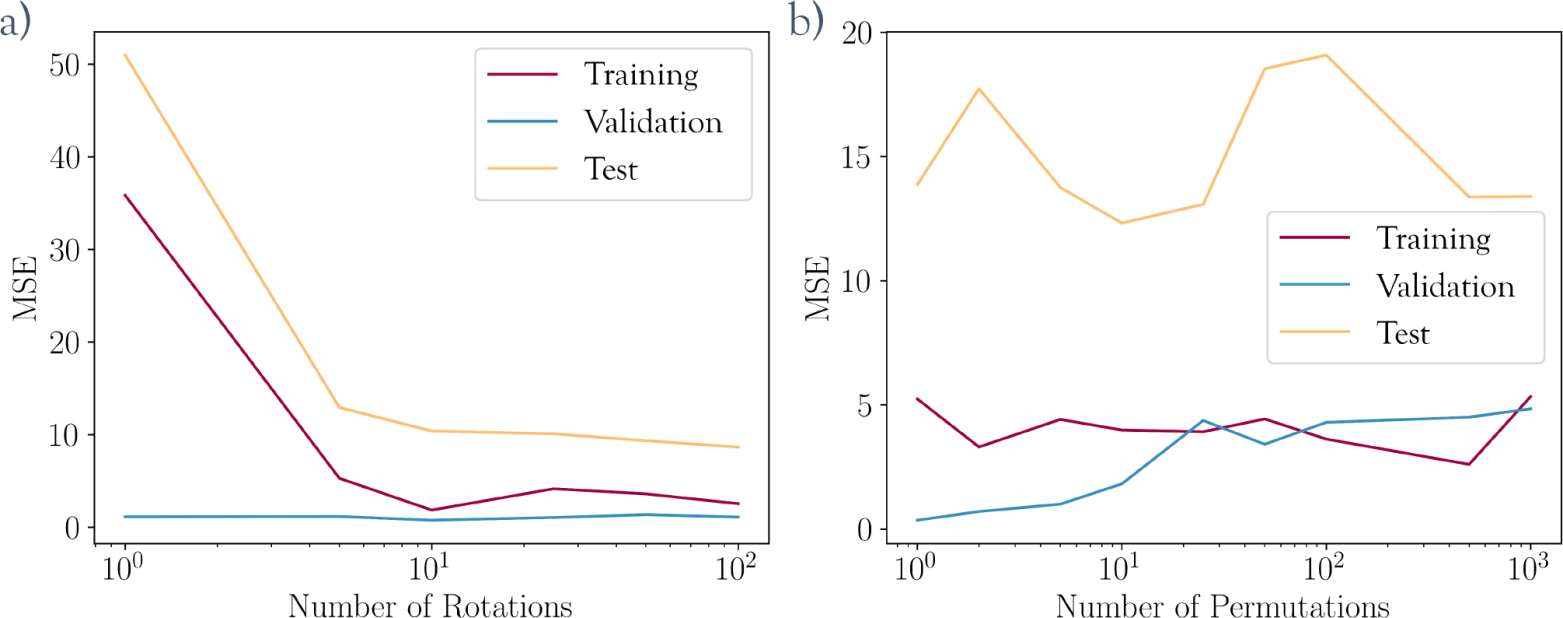
Learning invariances via brute-force data augmentation. (a) Performance of a model, expressed as mean square error (MSE), on a large set of randomly roto-translated structures (yellow) as a function of the number of rotated structures used at each training step. (b) Performance of a model on a large set of randomly permutated structures (yellow) as a function of the number of permutations used during each training step. In both panels, the red and blue lines show the training and validation set performances, respectively.

To test for this hypothesis, a model is trained on 80% of a nucleating trajectory with 10,000 frames using a loss function that evaluates the performance of a model on any given training frame and also over a set number of randomly generated roto-translated or permutated versions of that same frame. The remaining 20% are set aside as a hold-out validation set to monitor whether the model loses the ability to predict the CV on the base trajectory. A test set is created out of a large collection of randomly roto-translated frames. Figure 2 shows the performance of multiple models trained this way, including in the loss function an increasing number of evaluations over randomly roto-translated or permutated configurations. Each model had three layers of 512, 258, and 128 nodes, respectively. If the model can learn the underlying invariances, the expected behavior is to see the error over the test set slowly converge with the hold-out set error for an increasing number of loss function evaluations over roto-translations or permutations. This behavior can clearly be seen for the roto-translations (Figure 2a) but not for the permutations (Figure 2b). The only discernible trend in the permutations plot is the hold-out set error diverging with *n* since each training step becomes more and more diluted.

This observation demonstrates that a brute-force approach cannot efficiently learn all the invariances via a sufficiently large dataset. The most obvious reason for this failure is the vast number of possible permutations. For instance, in this application, the number of possible ways of inputting the 421 particles is practically infinite. However, so is the number of possible rotations that one can apply to any configuration. A significant difference, though, is that any infinitesimal change in rotation leads to an equivalent infinitesimal change in the input vector of the multilayer perceptron. Therefore, the hypersphere of possible quaternions corresponds to a smooth orbit of points in configuration space,^49^ and the shape of this orbit can be inferred from a limited number of data points. In contrast, each possible permutation corresponds to its unique input vector, with no differentiable path connecting them. This implies that to create a neural network that behaves as if it were permutationally invariant, one would need at least *n*! data points, a dataset size that is practically unachievable for any meaningful nucleation problem.

These results justify the switch to a more expensive, inherently permutationally invariant model architecture based on graph neural networks. Alternatively, one could replace the Cartesian coordinates with a permutationally invariant descriptor. However, a graph-based architecture has several additional advantages; most notably, its structure mirrors the evaluation of the analytical function that needs to be approximated with a pooling of local contributions. This locality of the model also makes it more data-efficient and robust outside of its training domain. Moreover, a graph-based architecture lends itself to be easily adapted to capture additional complexity, if necessary.

### Training of a GNN *n*(*Q*6) Approximator

#### Loss Function

The goal is to train models that facilitate the simulation of nucleation events by acting as CVs in the enhanced sampling approaches. However, a cheap error metric is required to train such a model to perform the loss minimization. Such an error metric is necessarily just a proxy for the actual quality of the model as a CV and should be considered as such. In this work, the loss function used during training is the mean square error (MSE).

#### Training Set

**Figure 3:**
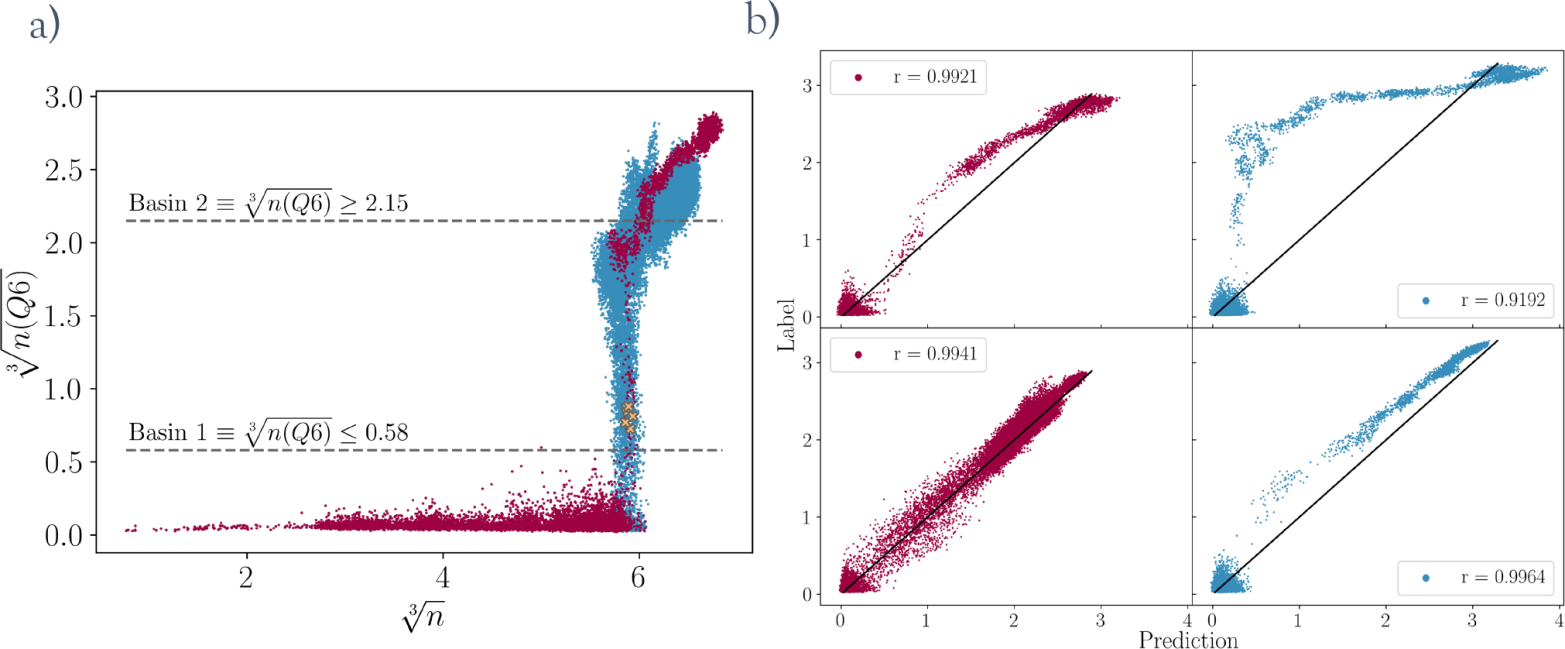
Enriching the dataset along poorly sampled transition regions. (a) Dataset (blue) generated from one initial trajectory (red) by restarting 67 short simulations from the configurations marked with yellow crosses until they reach one of the basins. (b) Performance of two representative models trained on just the initial trajectory (top) and the extended set (bottom), evaluated on their respective training sets (left) and an independent trajectory (right).

A baseline training set is constructed by supplementing a nucleating trajectory of 10k frames with additional transition state data from a committor analysis of the system (32k frames in total) (see Figure 3). When constructing a CV approximation for sampling rare events from unbiased data, one has to deal with the fact that by definition the transition state of interest will be underrepresented compared to the metastable states. Here the training data are supplemented by starting short bursts of simulations from configurations in the transition region until they reach one of the basins. This drastically improves the model’s accuracy in this region of CV space, as shown in Figure 3b.

**Figure 4:**
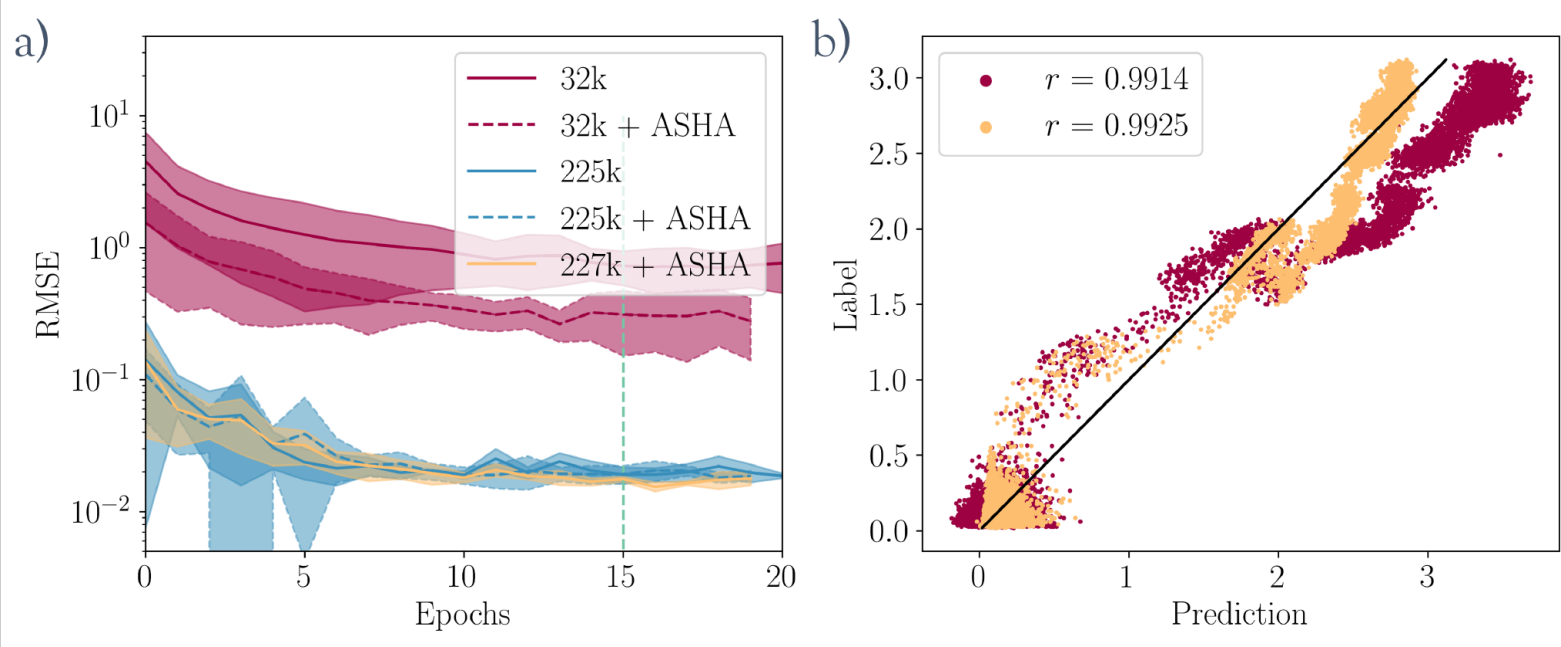
Optimizing the GNN model hyperparameters. (a) Average model convergence, with one standard deviation, of models trained on different datasets and different sets of hyperparameters. The different lines are labeled with the numbers of data points in the corresponding datasets and whether the hyperparameters were optimized (ASHA) or not. (b) Scatter of model predictions against the labels on an independent trajectory. The points are colored according to the training set of the corresponding model from (a).

A model trained on this dataset (red) (see Figure 4b) is capable of predicting  with almost perfect correlation (Pearson correlation coefficient *r* > 0.99) on a completely independent trajectory extracted from the ensemble of transition paths characteristic of the nucleation problem at hand. Importantly, the model shown does not misclassify a single frame as crystalline  when it is not. This, combined with the perfect correlation, makes the trained GNN model a suitable candidate to be used as a CV.

Figure 4a shows that optimizing the hyperparameters and massively expanding the dataset can improve the model accuracy and reduce the training variance. However, Figure 4b shows that most of the additional accuracy comes from improving the model accuracy at high values of  without meaningfully impacting the correlation of predictions. Therefore, to stay true to the application case, in which data are always scarce, the model trained on the small set is used for all the following biased simulations.

#### Test on an Independent Trajectory

As can be seen on the right side of Figure 4, graph-based models can be trained to accurately predict the value of *n*(*Q*6). The previously mentioned structural similarities between the GNN model and the analytical variable to be approximated allow a relatively small model to make highly accurate predictions, even outside of its training domain. To quantitatively support this observation, we report a systematic evaluation of the model hyperparameters. The yellow model in Figure 4 has a latent dimensionality of 8 and possesses two graph convolutional layers, totaling 960 parameters. Its local nature makes it much less sensitive to leaving the training domain as it estimates the influence of local environments on the final CV value. In fact, a sufficiently large training set can efficiently capture the structural diversity of local environments, which is significantly smaller than that of their combinations in global environments. Another factor that simplifies the learning process is that the models learn to predict the cube root of *n*(*Q*6) instead of *n*(*Q*6) directly. Physically, this value can be interpreted as the radius of the crystalline domain, but mainly it compresses the range of *n*(*Q*6) values around the transition state. The primary energy barrier of the system is around *n*(*Q*6) = 1, and the model needs to distinguish early-stage nuclei from liquid frames. Thus, taking the cube root reduces the influence of the model performance on large crystals relative to the more important transition state region during training. Therefore, the models used in this work are trained to predict  instead of *n*(*Q*6).

### Evaluating GNN Model Performances in Biased Simulations

**Figure 5:**
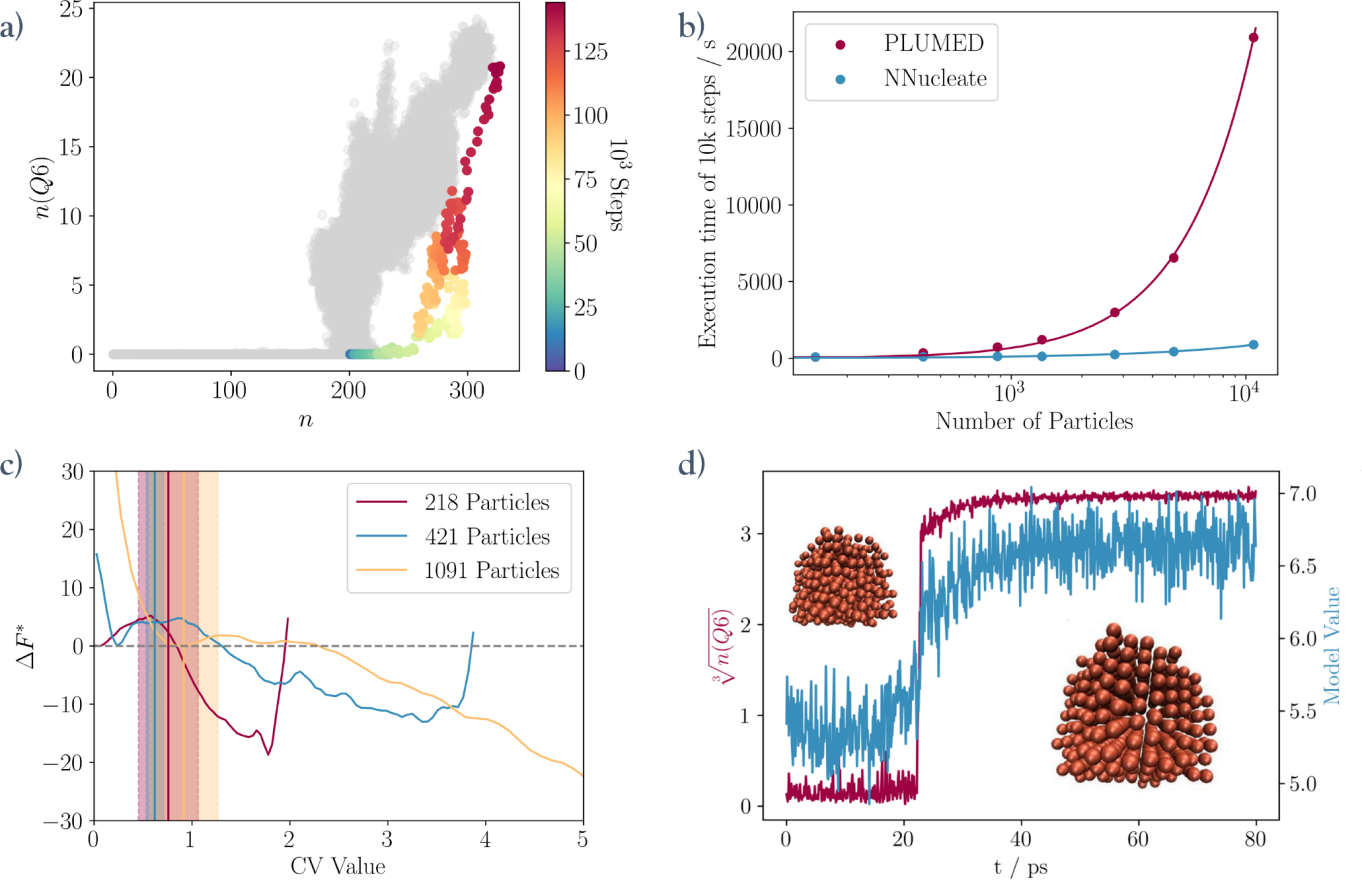
Deploying a GNN model in biased simulations. (a) CV values for a nucleating trajectory obtained in a pulling simulation using a model CV. The gray shading represents the training set of the GNN used in the simulation. (b) Scaling of the cost of performing 10k metadynamics steps using the method presented in this work (NNucleate) with system size compared to performing the same metadynamics steps with the explicit calculation of the CV in PLUMED. (c) Free energy profiles (reduced units) obtained with umbrella sampling on three systems of varying sizes. The vertical lines indicate the critical nucleus sizes with their corresponding errors upon reweighting those free energy surfaces to the reference collective variable. The critical nucleus size, as indicated by the position of the transition state, is estimated to be 0.76 ± 0.31 (218 particles), 0.62 ± 0.09 (421 particles), and 0.92 ± 0.35 (1091 particles). (d) CV values from a short pulling simulation in a separate system of 500 copper particles. This showcases the model’s ability to induce crystallization in a system different from the one it was trained in.

A way to test the suitability of a trained model as a CV is to perform a pulling or moving restraint simulation. This simulation “pulls” the system along a defined CV by applying a harmonic restraint that gradually moves throughout the simulation. Figure 5a shows the trajectory of such a simulation that was obtained by using the CV model highlighted in red in Figure 4 to pull the system through phase space. The system rapidly crosses the high energy barrier at , and the resulting nucleus grows into a crystalline domain. The figure also illustrates how the pulling simulation pushes the model far outside its training domain, demonstrating the model’s robustness and indicating that even outside its training domain, the model can still make predictions correlated to the true values of .

#### Scaling of Computational Costs

At the system size where training is performed (421 particles), the GNN CV model is 3.5 times more cost-effective at performing biased simulations than the reference  CV (see Figure 5b). While this is not overly impressive, considering the effort necessary to create such a model, the true power of this approach shows when moving to larger systems. Even in systems as simple as this one, the cost of performing biasing steps grows dramatically with system size, making simulations with a number of particles  virtually unfeasible. However, the model developed here showcases a much more favorable cost scaling. This is especially relevant since the model is size-transferable. The model can be trained at a size where reference data generation is feasible but then applied to much larger systems. This opens up new ways of studying large atomistic systems with enhanced sampling methods. It further suggests that the cost gain could be even larger when approximating more complex CVs, e.g., for molecular systems. It also answers the inherent “chicken-and-egg” problem in data-driven CVs. This means that to construct a CV from data, we need to sample relevant configurations, which are by definition hard to obtain; otherwise, CV-based sampling methods would not be needed in the first place. Adopting a GNN-based approach, one can train model CVs in a small system, where sampling is computationally accessible, and deploy them in larger systems.

#### Umbrella Sampling Simulations

A key area of application for these models is the generation of free energy profiles. Figure 5c shows three free energy profiles obtained using the same model in three different systems of various sizes via umbrella sampling. The free energy profile obtained for the 421 particle system exhibits all the expected features: a minimum for the dense liquid droplet is near 0, there is a barrier at around 1, and a crystalline basin exists at higher model CV values. The repulsive wall to the right of the free energy surface (FES) basin representing the crystalline state in the system results from finite-size effects. The other two profiles show similar shapes and features. However, the location of the nucleation barrier shifts with the system size. This is a problem, as the critical nucleus size should remain independent of the system size. However, the reason for this is that the model tends to overestimate *n*(*Q*6) values in larger systems and underestimate them in smaller systems. Looking at the training set of the model in Figure 5a, one can see that the droplet size is strongly correlated with *n*(*Q*6) beyond *n* ≈ 200. Thus, the model learns to associate larger droplets, even without order, with larger predictions, and the free energy profiles shift with the equilibrium size of the dense liquid droplet. However, this only minimally impacts the model’s ability to detect order. The height of the nucleation barrier, projected onto the model CV, matches across the small systems but decreases for the largest one. This can be partly explained by the fact that in a larger system, due to the nature of the CV, it is hard to restrict nucleation to a single site, and biasing a system-wide CV leads to multiple clusters emerging throughout the simulation box. For a better comparison of the FESs, they should be reweighted to the analytical CV. Here this is done using the weighted histogram analysis method (WHAM).^50^Figure 5c shows the critical nucleus sizes obtained from the reweighted surfaces as vertical lines. These values are obtained by averaging all CV values that fall within one standard deviation of the maximum of the reweighted FES, and the shading is the corresponding standard deviation. This leads to a consistent estimate of the critical nucleus size across all simulated systems, in agreement with an independent estimate of the critical nucleus size obtained from committor analysis.

These results demonstrate that the transferability of the model CV is sufficient to yield a physically consistent picture of the nucleation process across several system sizes. In its current implementation, the main limitation associated with simulating large systems is no longer the computational cost of the CV but rather its GPU memory requirements.

#### System Transferability

A GNN model that is truly able to capture the local structure of particle environments approximating the collective variable *n*(*Q*6) should be applicable to other systems in which the crystallization process can also be described by *n*(*Q*6). To test system transferability, a copper melt is created by equilibrating a 500-atom copper fcc crystal at 200 K for 1 ns at a fixed pressure of 1 bar and then melting it by heating it from 200 to 2000 K over the course of 5 ns. Finally, the melt is supercooled back down to 1100 K for 7.5 ns.

Figure 5d shows the result of a pulling simulation performed with the same model and the same parameters as the one in Figure 5a. The only change made to the model is that the cutoff radius for the neighbor list generation for the model input is adapted to the radial distribution function of the copper crystal. Evidently, the model can still describe the nucleation process by distinguishing liquid and crystal-like configurations. As such, it can be used as a pulling CV to drive crystallization from the copper melt. The model was able to achieve this even though it never encountered a metal melt in its training. In fact, it was trained on configurations sampling three metastable states, including a dispersed phase, a dense liquid, and a crystal. In contrast, the nucleation of a metal from its melt involves only two metastable states. Importantly, in the training set, local density and local order show a degree of correlation, while in the case of the copper melt, order emerges without system-wide density fluctuations. We are convinced that this is possible because the model has learned to capture the local structure of a crystal in a way that strongly resembles the analytical CV. We explicitly interrogate this assertion in the following section. Finally, system transferability further justifies the computational resources required to construct and train a GNN model CV and opens up additional avenues for its application.

#### Limitations and Critical Assessment of Transferability

Despite the success in achieving accurate and transferable predictions, the method presented has inherent limitations. As previously mentioned, the training set contains the bias that high values of *n* correlate with larger values of *n*(*Q*6), which, for instance, leads to a high baseline prediction in the copper melt (see Figure 5d). This, combined with the higher noise level in the model predictions caused by the structural differences between the copper melt configurations and the training data, reduces the resolution in the classification of metastable states.

These effects limit the simultaneous size and system transferability of the model CV, for which we can foresee a size limit where it loses the ability to distinguish between the crystalline and melt configurations.

**Figure 6:**
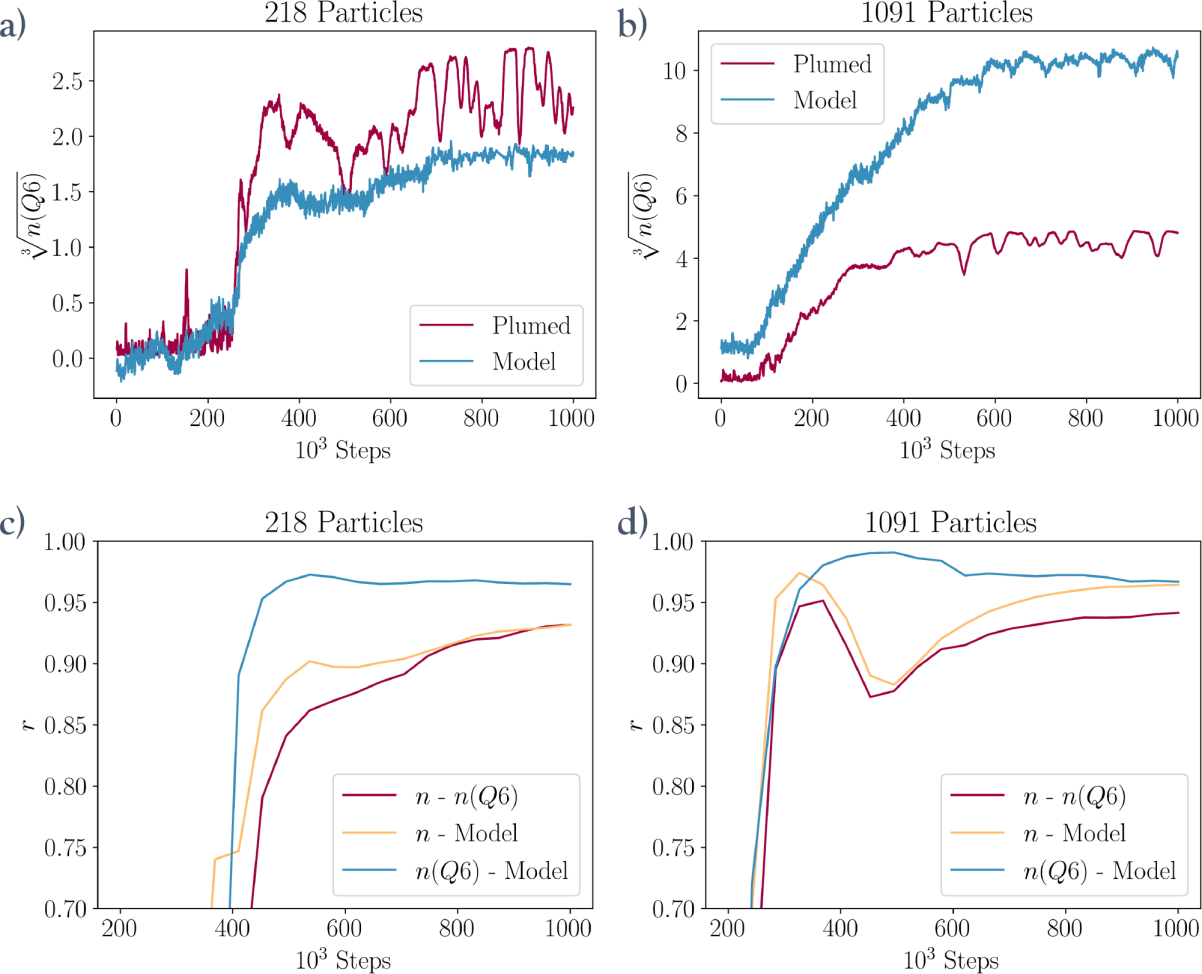
(a, b) Model predictions and reference values throughout two pulling simulations performed by the same model in a small system and a large system. (c, d) Plots showing how the total correlation between the model, *n*, and *n*(*Q*6) over all frames up to the indicated step count evolves throughout the simulation.

The successes of the model in the copper melt suggest that it is capable of distinguishing between local order and disorder regardless of local density, yet it is important to critically analyze whether the model in larger systems still actually facilitates ordering or just the formation of a larger and larger dense liquid droplet. To this aim, we analyze the correlation between the order and density in the model CV and the corresponding analytical reference. The top row in Figure 6 shows two pulling simulations using the same model CV starting from small dense liquid droplets in two different-sized systems. In the small system (see Figure 6a,c), the initial steps of the simulation are characterized by the dense liquid droplet growing until it reaches its equilibrium size. This process is followed by the crossing of the nucleation barrier, which coincides with a sharp increase in *n*(*Q*6). As expected, the model predictions increase at exactly the same time as the reference values. However, in the large system, the model values increase around 40,000 steps earlier than the reference values (Figure 6b). This increase is due to the growth of the dense liquid droplet far beyond the sizes represented in the training set. The yellow line in Figure 6d indicates that in this range the model is most closely correlated to the variable *n* and not *n*(*Q*6). After this initial period, the droplet stops growing, and the crystalline domain emerges. At this stage, the model goes back to being perfectly correlated to the reference *n*(*Q*6) values.

This increase in “*n* character” with increasing system size is not inherently a problem and is unique to this combination of system and CVs. It does, however, serve as a reminder that this is fundamentally a machine-learning approach and, as such, it is limited by its training dataset. Therefore, any new insights into nucleation mechanisms obtained using this approach should be critically analyzed through the lens of dataset biases. Fortunately, there are plenty of ways to supplement and manipulate training datasets with collected or synthesized data to combat such biases.

#### Constructing Multivariate Models for Adaptive Biasing in Two Dimensions

So far, in this work we have highlighted different aspects of how the approximative power of the presented framework can be leveraged into computational efficiency gains in enhanced sampling applications. However, another way of increasing these gains is by approximating more than one CV at once. In the models discussed up to now, the final graph decoding layer maps the *m*-dimensional internal vector representation of the model to a scalar value that is used as a CV. This, however, is a somewhat arbitrary choice. By changing the dimensionality of the output, the model can be trained to approximate multiple CVs at once at effectively the same computational cost.

**Figure 7:**
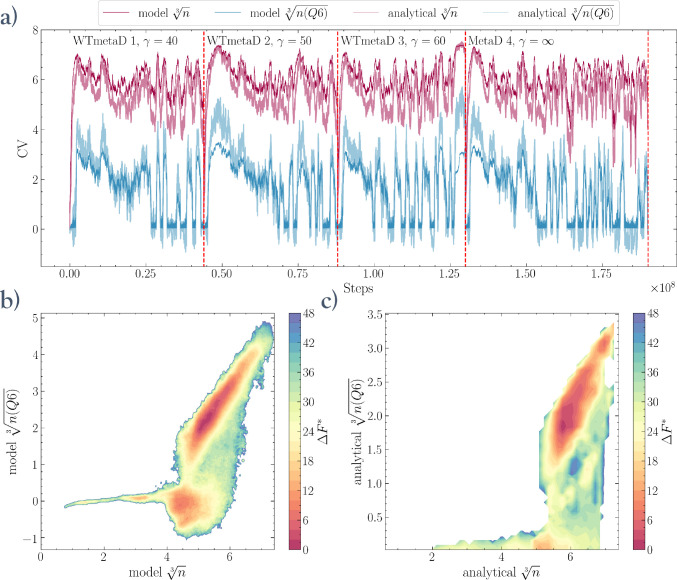
Four independent metadynamics simulations with different simulation protocols, including well-tempered metadynamics (WTmetaD) with different bias factors γ and standard metadynamics (MetaD), corresponding to γ = *∞*. In every simulation, the bias potential is computed in the space of two CVs ( and ) simultaneously predicted by a GNN model. All simulation setups reach a quasi-diffusive regime after the first recrossing between the liquid intermediate and the crystal states. (a) History of the CV values during the simulations. (b) Resulting free energy surface in the space of the *model* CVs computed merging the samples from all simulations with mean force integration (MFI).^51^ (c) Free energy surface function of the *analytical* CVs, which is recovered via reweighting. Additional details on MFI and the associated reweighting procedure are reported in the Supporting Information.^15,51−54^

In Figure 7, we demonstrate the application of one such model, trained to simultaneously predict the  and  CVs.^30^ We deployed this method to perform four independent two-dimensional metadynamics simulations in this CV space. Three of these simulations are well-tempered metadynamics (WTmetaD) with varying bias factors γ, while the fourth is a *standard*, nontempered metadynamics simulation. The combined sampling history of the four simulations is reported in Figure 7a. Here we can see that after a first recrossing, all four simulations reach a regime in which transitions between the metastable liquid and crystalline states can be efficiently and reversibly sampled. The two-dimensional FES obtained by combining the statistics of all four simulations using mean force integration (MFI)^51,55^ is shown in Figure 7b. This FES exhibits all of the expected features, with a basin for the dense liquid droplet and a deeper basin for the crystalline domain separated by a free energy barrier at *n*(*Q*6) ≈ 1. However, due to the statistical nature of the approximation provided by the model CVs, the resulting free energy surface is not an exact match to the one that an equivalent metadynamics simulation using the analytical variables would produce. The statistical noise in the CV prediction is shown by the time series of the analytical and model CVs reported in Figure 7a. Its effects are best exemplified in the liquid droplet basin, which extends into the negative values due to the larger fluctuations of the GNN-approximated variable compared to its analytical counterpart. This effect, however, does not hinder the exploration of the model configuration space and can be remedied by reweighting the FES back into the space of the analytical CVs. Here we obtain a reweighted FES as a function of the analytical  and  by combining samples from all four metadynamics simulations with time-independent weights computed via MFI.^15,51−54^

Moreover, reweighting is a postprocessing procedure entailing minimal computational effort, as it only requires the evaluation of the analytical variables every few hundred steps without the need for any gradients. The reweighted FES is reported in Figure 7b. Additional details on the four independent metadynamics simulations, together with further discussion of the reweighing method, are reported in the Supporting Information.

The model’s ability to accurately predict multiple variables simultaneously, in this case,  and , hints that we are possibly far from exhausting the full approximative potential of the approach described in this article, which we will further investigate in a dedicated follow-up publication.

## Conclusions

The framework developed in this work represents a powerful general approach to mapping the Cartesian coordinates of a system to its corresponding CV values. By sidestepping the calculation of expensive symmetry functions or similar local descriptors commonly used in comparable machine-learning approaches, we unlocked considerable gains in computational efficiency. This paves the way for the development of generally applicable approaches to enhance the sampling of nucleation events in complex systems that are currently out of reach, such as molecular crystals from solution.

The proposed graph-based architecture enforces permutational invariances and allows the model CV to learn rotational and translational invariances from data. Furthermore, such models are inherently size-transferable, which enables one to train the model at computationally accessible system sizes and deploy them in larger-scale simulations with minimal computational overhead.

In principle, due to its modular nature, the model presented here can be adapted to meet the demands of more complex CVs, such as those necessary when simulating nucleation in molecular systems.^21,24,36^

Solving the computational bottleneck associated with evaluating complex CVs in self-assembling systems is central to developing general approaches to studying nucleation. Thus, we are convinced that approaches like the one proposed here will be crucial to model crystallization in realistic environments.

## Data Availability

The input parameters to reproduce the enhanced sampling simulations in PLUMED can be found on PLUMED-NEST under the ID plumID:23.026 (https://www.plumed-nest.org/).^38^ The package to reproduce the GNN training procedures can be downloaded from https://github.com/mme-ucl/NNucleate. The same repository also contains the model parameters used to perform the simulations in this work. An implementation of the MFI method used to compute a joint free energy surface from multiple metadynamics simulations can be obtained at https://github.com/mme-ucl/MFI.

## Supporting Information Available

Additional details and figures related to the reweighting methods and FES obtained from 2D metadynamics simulations (PDF)

## References

[ref1] Price S. L. Control and prediction of the organic solid state: a challenge to theory and experiment. Proc. R. Soc. A 2018, 474, 20180351 10.1098/rspa.2018.0351. 30333710 PMC6189584

[ref2] Price S. L. Predicting crystal structures of organic compounds. Chem. Soc. Rev. 2014, 43, 2098–2111. 10.1039/C3CS60279F. 24263977

[ref3] Day G. M. Current approaches to predicting molecular organic crystal structures. Crystallogr. Rev. 2011, 17, 3–52. 10.1080/0889311X.2010.517526.

[ref4] Sun C. C.; Sun W.; Price S.; Hughes C.; Ter Horst J.; Veesler S.; Lewtas K.; Myerson A.; Pan H.; Coquerel G.; et al. Solvent and additive interactions as determinants in the nucleation pathway: general discussion. Faraday Discuss. 2015, 179, 383–420. 10.1039/C5FD90038G. 26083497

[ref5] Anderson M. W.; Bennett M.; Cedeno R.; Cölfen H.; Cox S. J.; Cruz-Cabeza A. J.; De Yoreo J. J.; Drummond-Brydson R.; Dudek M. K.; Fichthorn K. A.; et al. Understanding crystal nucleation mechanisms: where do we stand? General discussion. Faraday Discuss. 2022, 235, 219–272. 10.1039/D2FD90021A. 35789238

[ref6] Price S.; Rimez B.; Sun W.; Peters B.; Christenson H.; Hughes C.; Sun C. C.; Veesler S.; Pan H.; Brandel C.; et al. Nucleation in complex multi-component and multi-phase systems: general discussion. Faraday Discuss. 2015, 179, 503–542. 10.1039/C5FD90039E. 26081969

[ref7] Giberti F.; Salvalaglio M.; Parrinello M. Metadynamics studies of crystal nucleation. IUCr J. 2015, 2, 256–266. 10.1107/S2052252514027626. PMC4392418 25866662

[ref8] Finney A.; Salvalaglio M. Theoretical and computational approaches to study crystal nucleation from solution. ChemRxiv 2023, 10.26434/chemrxiv-2023-rb79v.

[ref9] Sosso G. C.; Chen J.; Cox S. J.; Fitzner M.; Pedevilla P.; Zen A.; Michaelides A. Crystal Nucleation in Liquids: Open Questions and Future Challenges in Molecular Dynamics Simulations. Chem. Rev. 2016, 116, 7078–7116. 10.1021/acs.chemrev.5b00744. 27228560 PMC4919765

[ref10] Agarwal V.; Peters B. Solute precipitate nucleation: A review of theory and simulation advances. Adv. Chem. Phys. 2014, 155, 97–160. 10.1002/9781118755815.ch03.

[ref11] Peters B. Reaction Rate Theory and Rare Events; Elsevier, 2017.

[ref12] Allen R. J; Valeriani C.; Rein ten Wolde P. Forward flux sampling for rare event simulations. J. Phys.: Condens. Matter 2009, 21, 463102 10.1088/0953-8984/21/46/463102. 21715864

[ref13] Jiang H.; Haji-Akbari A.; Debenedetti P. G.; Panagiotopoulos A. Z. Forward flux sampling calculation of homogeneous nucleation rates from aqueous NaCl solutions. J. Chem. Phys. 2018, 148, 044505 10.1063/1.5016554. 29390820

[ref14] Hall S. W.; Díaz Leines G.; Sarupria S.; Rogal J. Practical guide to replica exchange transition interface sampling and forward flux sampling. J. Chem. Phys. 2022, 156, 200901 10.1063/5.0080053. 35649875

[ref15] Torrie G.; Valleau J. Nonphysical sampling distributions in Monte Carlo free-energy estimation: Umbrella sampling. J. Comput. Phys. 1977, 23, 187–199. 10.1016/0021-9991(77)90121-8.

[ref16] Laio A.; Parrinello M. Escaping free-energy minima. Proc. Natl. Acad. Sci. U. S. A. 2002, 99, 12562–12566. 10.1073/pnas.202427399. 12271136 PMC130499

[ref17] Barducci A.; Bussi G.; Parrinello M. Well-tempered metadynamics: a smoothly converging and tunable free-energy method. Phys. Rev. Lett. 2008, 100, 020603 10.1103/PhysRevLett.100.020603. 18232845

[ref18] Darve E.; Rodríguez-Gómez D.; Pohorille A. Adaptive biasing force method for scalar and vector free energy calculations. J. Chem. Phys. 2008, 128, 144120 10.1063/1.2829861. 18412436

[ref19] Marchi M.; Ballone P. Adiabatic bias molecular dynamics: a method to navigate the conformational space of complex molecular systems. J. Chem. Phys. 1999, 110, 3697–3702. 10.1063/1.478259.

[ref20] Hénin J.; Lelièvre T.; Shirts M. R.; Valsson O.; Delemotte L. Enhanced sampling methods for molecular dynamics simulations. arXiv (Condensed Matter > Statistical Mechanics), August 25, 2022, 2202.04164, ver. 2. https://arxiv.org/abs/2202.04164 (accessed 2023-08-18).

[ref21] Santiso E. E.; Trout B. L. A General Set of Order Parameters for Molecular Crystals. J. Chem. Phys. 2011, 134, 064109 10.1063/1.3548889. 21322663

[ref22] Neha; Tiwari V.; Mondal S.; Kumari N.; Karmakar T. Collective Variables for Crystallization Simulationsfrom Early Developments to Recent Advances. ACS Omega 2023, 8, 127–146. 10.1021/acsomega.2c06310. 36643553 PMC9835087

[ref23] Steinhardt P. J.; Nelson D. R.; Ronchetti M. Bond-orientational order in liquids and glasses. Phys. Rev. B 1983, 28, 784–805. 10.1103/PhysRevB.28.784.

[ref24] Gobbo G.; Bellucci M. A.; Tribello G. A.; Ciccotti G.; Trout B. L. Nucleation of Molecular Crystals Driven by Relative Information Entropy. J. Chem. Theory Comput. 2018, 14, 959–972. 10.1021/acs.jctc.7b01027. 29272581

[ref25] Gimondi I.; Salvalaglio M. CO2 Packing Polymorphism Under Confinement in Cylindrical Nanopores. Mol. Syst. Des. Eng. 2018, 3, 243–252. 10.1039/C7ME00103G.

[ref26] Giberti F.; Salvalaglio M.; Mazzotti M.; Parrinello M. Insight into the nucleation of urea crystals from the melt. Chem. Eng. Sci. 2015, 121, 51–59. 10.1016/j.ces.2014.08.032.

[ref27] Piaggi P. M.; Parrinello M. Predicting polymorphism in molecular crystals using orientational entropy. Proc. Natl. Acad. Sci. U. S. A. 2018, 115, 10251–10256. 10.1073/pnas.1811056115. 30237287 PMC6187181

[ref28] Schran C.; Brezina K.; Marsalek O. Committee neural network potentials control generalization errors and enable active learning. J. Chem. Phys. 2020, 153, 104105 10.1063/5.0016004. 32933264

[ref29] Peters B. Reaction Coordinates and Mechanistic Hypothesis Tests. Annu. Rev. Phys. Chem. 2016, 67, 669–690. 10.1146/annurev-physchem-040215-112215. 27090846

[ref30] Finney A. R.; Salvalaglio M. A variational approach to assess reaction coordinates for two-step crystallization. J. Chem. Phys. 2023, 158, 094503 10.1063/5.0139842. 36889939

[ref31] Zou Z.; Beyerle E. R.; Tsai S.-T.; Tiwary P. Driving and characterizing nucleation of urea and glycine polymorphs in water. Proc. Natl. Acad. Sci. U. S. A. 2023, 120, e2216099120 10.1073/pnas.2216099120. 36757888 PMC9963467

[ref32] Kipf T. N.; Welling M. Semi-Supervised Classification with Graph Convolutional Networks. arXiv (Computer Science.Machine Learning), February 22, 2017, 1609.02907, ver. 4. https://arxiv.org/abs/1609.02907 (accessed 2023-08-18).

[ref33] Defferrard M.; Bresson X.; Vandergheynst P. Convolutional Neural Networks on Graphs with Fast Localized Spectral Filtering. arXiv (Computer Science.Machine Learning), February 5, 2017, 1606.09375, ver. 3. https://arxiv.org/abs/1606.09375 (accessed 2023-08-18).

[ref34] Thompson A. P.; Aktulga H. M.; Berger R.; Bolintineanu D. S.; Brown W. M.; Crozier P. S.; in’t Veld P. J.; Kohlmeyer A.; Moore S. G.; Nguyen T. D.; et al. LAMMPS-a flexible simulation tool for particle-based materials modeling at the atomic, meso, and continuum scales. Comput. Phys. Commun. 2022, 271, 108171 10.1016/j.cpc.2021.108171.

[ref35] ten Wolde P. R.; Frenkel D. Computer simulation study of gas–liquid nucleation in a Lennard-Jones system. J. Chem. Phys. 1998, 109, 9901–9918. 10.1063/1.477658.

[ref36] Tribello G. A.; Giberti F.; Sosso G. C.; Salvalaglio M.; Parrinello M. Analyzing and Driving Cluster Formation in Atomistic Simulations. J. Chem. Theory Comput. 2017, 13, 1317–1327. 10.1021/acs.jctc.6b01073. 28121147

[ref37] Tribello G. A.; Bonomi M.; Branduardi D.; Camilloni C.; Bussi G. PLUMED 2: New feathers for an old bird. Comput. Phys. Commun. 2014, 185, 604–613. 10.1016/j.cpc.2013.09.018.

[ref38] Bonomi M.; Bussi G.; Camilloni C.; Tribello G. A.; Banáš P.; Barducci A.; Bernetti M.; Bolhuis P. G.; Bottaro S.; Branduardi D.; et al. Promoting transparency and reproducibility in enhanced molecular simulations. Nat. Methods 2019, 16, 670–673. 10.1038/s41592-019-0506-8. 31363226

[ref39] Paszke A.; Gross S.; Massa F.; Lerer A.; Bradbury J.; Chanan G.; Killeen T.; Lin Z.; Gimelshein N.; Antiga L.; PyTorch: An Imperative Style, High-Performance Deep Learning Library. In Advances in Neural Information Processing Systems 32 (NeurIPS 2019); Curran Associates, 2019; pp 8024–8035.

[ref40] McGibbon R. T.; Beauchamp K. A.; Harrigan M. P.; Klein C.; Swails J. M.; Hernández C. X.; Schwantes C. R.; Wang L.-P.; Lane T. J.; Pande V. S. MDTraj: A Modern Open Library for the Analysis of Molecular Dynamics Trajectories. Biophys. J. 2015, 109, 1528–1532. 10.1016/j.bpj.2015.08.015. 26488642 PMC4623899

[ref41] Michaud-Agrawal N.; Denning E. J.; Woolf T. B.; Beckstein O. MDAnalysis: A toolkit for the analysis of molecular dynamics simulations. J. Comput. Chem. 2011, 32, 2319–2327. 10.1002/jcc.21787. 21500218 PMC3144279

[ref42] Gowers R. J.; Linke M.; Barnoud J.; Reddy T. J. E.; Melo M. N.; Seyler S. L.; Domański J.; Dotson D. L.; Buchoux S.; Kenney I. M.; Beckstein O. MDAnalysis: A Python Package for the Rapid Analysis of Molecular Dynamics Simulations. In Proceedings of the 15th Python in Science Conference (SciPy 2016), 2016; pp 98–105.

[ref43] Giorgino T. PYCV: a PLUMED 2 Module Enabling the Rapid Prototyping of Collective Variables in Python. J. Open Source Software 2019, 4, 1773 10.21105/joss.01773.

[ref44] Bradbury J.; Frostig R.; Hawkins P.; Johnson M. J.; Leary C.; Maclaurin D.; Necula G.; Paszke A.; VanderPlas J.; Wanderman-Milne S.; Zhang Q. JAX: composable transformations of Python+NumPy programs, 2018. http://github.com/google/jax (accessed 2023-08-18).

[ref45] Heek J.; Levskaya A.; Oliver A.; Ritter M.; Rondepierre B.; Steiner A.; van Zee M. Flax: A neural network library and ecosystem for JAX, 2020. http://github.com/google/flax (accessed 2023-08-18).

[ref46] Kingma D. P.; Ba J. Adam: A Method for Stochastic Optimization. arXiv (Computer Science.Machine Learning), January 30, 2017, 1807.05118, ver. 9. https://arxiv.org/abs/1412.6980 (accessed 2023-08-18)

[ref47] Li L.; Jamieson K.; Rostamizadeh A.; Gonina K.; Hardt M.; Recht B.; Talwalkar A. Massively Parallel Hyperparameter Tuning, 2018; https://openreview.net/forum?id=S1Y7OOlRZ (accessed 2023-08-18).

[ref48] Liaw R.; Liang E.; Nishihara R.; Moritz P.; Gonzalez J. E.; Stoica I. Tune: A Research Platform for Distributed Model Selection and Training. arXiv (Computer Science.Machine Learning), July 13, 2018, 1807.05118, ver. 1. https://arxiv.org/abs/1807.05118 (accessed 2023-08-18).

[ref49] Finzi M.; Stanton S.; Izmailov P.; Wilson A. G. Generalizing Convolutional Neural Networks for Equivariance to Lie Groups on Arbitrary Continuous Data. arXiv (Statistics.Machine Learning), September 24, 2020, 2002.12880, ver. 3. https://arxiv.org/abs/2002.12880 (accessed 2023-08-18).

[ref50] Roux B. The calculation of the potential of mean force using computer simulations. Comput. Phys. Commun. 1995, 91, 275–282. 10.1016/0010-4655(95)00053-I.

[ref51] Marinova V.; Salvalaglio M. Time-independent free energies from metadynamics via mean force integration. J. Chem. Phys. 2019, 151, 164115 10.1063/1.5123498. 31675889

[ref52] Zwanzig R. W. High-temperature equation of state by a perturbation method. I. Nonpolar gases. J. Chem. Phys. 1954, 22, 1420–1426. 10.1063/1.1740409.

[ref53] Bonomi M.; Barducci A.; Parrinello M. Reconstructing the equilibrium Boltzmann distribution from well-tempered metadynamics. J. Comput. Chem. 2009, 30, 1615–1621. 10.1002/jcc.21305. 19421997

[ref54] Tiwary P.; Parrinello M. A time-independent free energy estimator for metadynamics. J. Phys. Chem. B 2015, 119, 736–742. 10.1021/jp504920s. 25046020

[ref55] Kästner J.; Thiel W. Bridging the gap between thermodynamic integration and umbrella sampling provides a novel analysis method: “Umbrella integration”. J. Chem. Phys. 2005, 123, 144104 10.1063/1.2052648. 16238371

