# Supplementary material for: Machine Learning Nucleation Collective Variables with Graph Neural Networks
Source: J Chem Theory Comput. 2023 Oct 25;20(4):1600–11. doi: 10.1021/acs.jctc.3c00722 (PMC10902841; doi:10.1021/acs.jctc.3c00722)
Supplement: Supplementary file 1 — ct3c00722_si_001.pdf [file ct3c00722_si_001.pdf]

Supplementary Information: Machine Learning Nucleation Collective Variables with Graph Neural Networks

Florian M. Dietrich,[†] Xavier R. Advincula,[†,¶] Gianpaolo Gobbo,[‡] Michael A. Bellucci,[‡] and Matteo Salvalaglio[*,†]

†*Thomas Young Centre and Department of Chemical Engineering, University College London, London WC1E 7JE, UK*

‡*XtalPi Inc, 245 Main Street, Cambridge, Massachusetts 02142, United States*

¶*Current address: Yusuf Hamied Department of Chemistry, University of Cambridge, Lensfield Road, Cambridge, CB2 1EW, UK*

E-mail: m.salvalaglio@ucl.ac.uk

Computing Free Energy Surfaces with Mean Force Integration

In order to merge the sampling obtained from independent metadynamics simulations and obtain a single free energy surface (FES), we apply Mean Force Integration (MFI).[1] With MFI, we estimate the mean force in Collective Variable (CV) space $\mathbf{s}$ used for constructing and applying the bias potential $V(\mathbf{s})$, and following the approach introduced by Kastner with Umbrella Integration,[2] we obtain the free energy surface by integrating the mean force in $\mathbf{s}$. Here $\mathbf{s}$ is composed of the graph-based approximation of $\sqrt[3]{n}$ and $\sqrt[3]{n(Q6)}$ as defined in the main manuscript. In a simulation where the bias potential $V_t(\mathbf{s})$ is updated at time intervals τ, the mean force in $\mathbf{s}$ is:

$$\nabla F_t(\mathbf{s}) = -\nabla\beta^{-1}\ln p_t^b(\mathbf{s}) - \nabla V_t(\mathbf{s}) = \begin{pmatrix} -\frac{\partial\beta^{-1}\ln p_t^b(\sqrt[3]{n})}{\partial\sqrt[3]{n}} - \frac{\partial V_t(\sqrt[3]{n},\sqrt[3]{n(Q6)})}{\partial\sqrt[3]{n}} \\ -\frac{\partial\beta^{-1}\ln p_t^b(\sqrt[3]{n(Q6)})}{\partial\sqrt[3]{n(Q6)}} - \frac{\partial V_t(\sqrt[3]{n},\sqrt[3]{n(Q6)})}{\partial\sqrt[3]{n(Q6)}} \end{pmatrix} \tag{S1}$$

where $\nabla F_t(\mathbf{s})$ is the mean force in $\mathbf{s}$, $p_t^b(\mathbf{s})$ is the biased probability density sampled in the time interval of length τ, beginning at time t, and $\beta = (k_B T)^{-1}$ with k_B being the Boltzmann constant, and T the temperature. In order to merge the force contributions generated by iterative, history-dependent updates of a metadynamics bias potential into a single estimate of the force in $\mathbf{s}$, we adopt a weighted sum, as proposed in the Umbrella Integration method[1,2]:

$$\left\langle \nabla F_t(\mathbf{s}) \right\rangle = \frac{\sum_{t'=1}^{t} p_{t'}^b(\mathbf{s})\nabla F_{t'}(\mathbf{s})}{\sum_{t'=1}^{t} p_{t'}^b(\mathbf{s})} \tag{S2}$$

where the biased probability density $p_t^b(\mathbf{s})$ is used as a position-dependent weight function to localise the estimate of the force in the region of $\mathbf{s}$ sampled in the time interval $[t; t+\tau]$. The FES in $\mathbf{s}$ is computed by numerically integrating the mean force $\langle\nabla F_t(\mathbf{s})\rangle_t$. In Figure S1, on the left column, we report for all the metadynamics simulations performed in this work, the FES reconstructed by integrating the mean force in the space of the GNN-predicted CVs, indicated as *model n*, and $n(Q6)$, respectively.

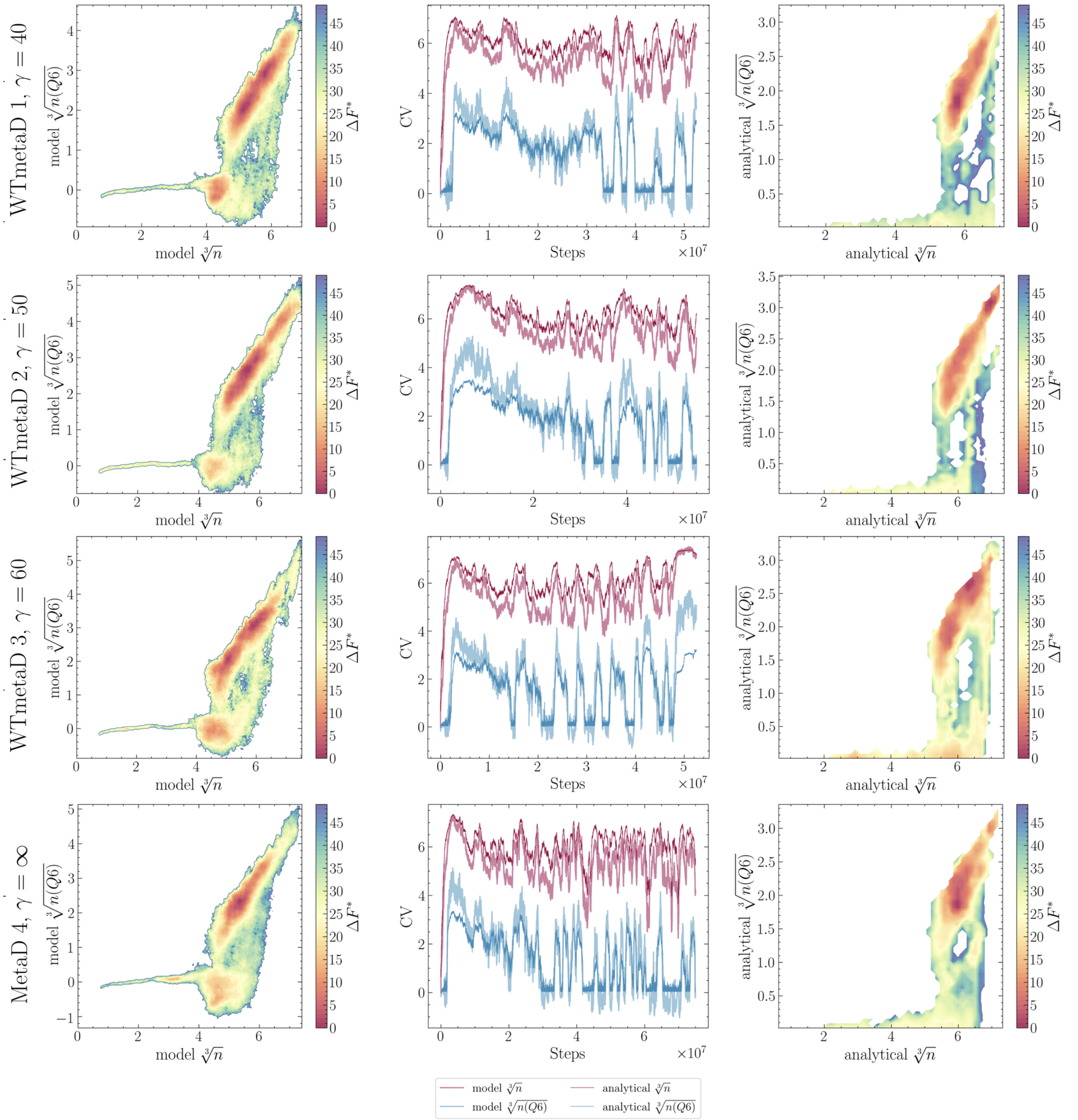

Figure S1: Sampling and Free Energy Surfaces from Well-Tempered and standard metadynamics. The *left* column reports FES computed as a function of the *biased* collective variables, namely the GNN-approximated n and $n(Q6)$. The *central* column shows the time series of both model (light color) and analytical (dark color) collective variables. Introducing a bias potential function of the GNN-approximated variables (the *model* CVs), enables a reversible sampling of transitions between the liquid-droplet and crystal states. The *right* column displays reweighted free energy surfaces as a function of the analytical CVs, which are computed a-posteriori every 500 steps at a negligible overhead cost. All simulations setup tested achieve reversible sampling.

Reweighting to obtain an FES function of the analytical CVs

As discussed in the main manuscript, the statistical nature of the GNN prediction introduces noise, and in order to recover an FES in the space of the analytical CVs, one can post-process a trajectory by reweighting.[3–6] Here, to perform reweighting, we take advantage of the approach outlined in Marinova and Salvalaglio[1], where it is noted that integrating the mean force decouples the calculation of the free energy surface $F(\mathbf{s})$ from the calculation of the time-dependent component of the metadynamics bias $c(t)$.[5,6] Hence we perform reweighting by assigning to any configuration extracted at time t from a given biased trajectory the weight:

$$w_t = e^{\beta(V_t(\mathbf{s})-c(t))} \tag{S3}$$

where $V_t(\mathbf{s})$ is the instantaneous total bias potential acting in the biased CVs $\mathbf{s}$, and $c(t)$ is computed from its definition as:

$$c(t) = \beta^{-1} \ln \frac{\int e^{-F(\mathbf{s})d\mathbf{s}}}{\int e^{-\beta[F(\mathbf{s})+V_t(\mathbf{s})]d\mathbf{s}}} \tag{S4}$$

where $F(\mathbf{s})$ is estimated from the integral of the mean thermodynamic force $\left\langle \nabla F_t(\mathbf{s}) \right\rangle_t$ (Eq. S2). This approach to reweighting applies to both Well-tempered and standard metadynamics and, therefore, can be applied to postprocess all the simulations performed to validate the applicability of the GNN-model CVs to metadynamics calculations. In Figure S1, central column, we report the time series of the *analytical* and *model* CVs showing that, despite showing an excellent correlation, statistical noise prevents them from perfectly matching. On the right column of the same figure, we display the FES function of the analytical CVs obtained by reweighting from each of the independent simulations performed in this study.

The setup of the four independent simulations performed in this study is summarized in Table S1, the corresponding PLUMED[7] input files are available in PLUMED-NEST (`https://www.plumed-nest.org/`, plumID:23.026[8]).

Table S1: Settings for the time-evolution of the bias potential $V_t(\mathbf{s})$ in metadynamics simulations 1-4, analysed in Fig S1.

Simulation	Algorithm	Height	σ_n	$\sigma_{n(Q6)}$	Pace	Bias Factor
1	Well-Tempered	0.1	0.18	0.05	1000	40
2	Well-Tempered	0.1	0.18	0.05	1000	50
3	Well-Tempered	0.1	0.18	0.05	1000	60
4	Standard	0.1	0.18	0.05	1000	N/A

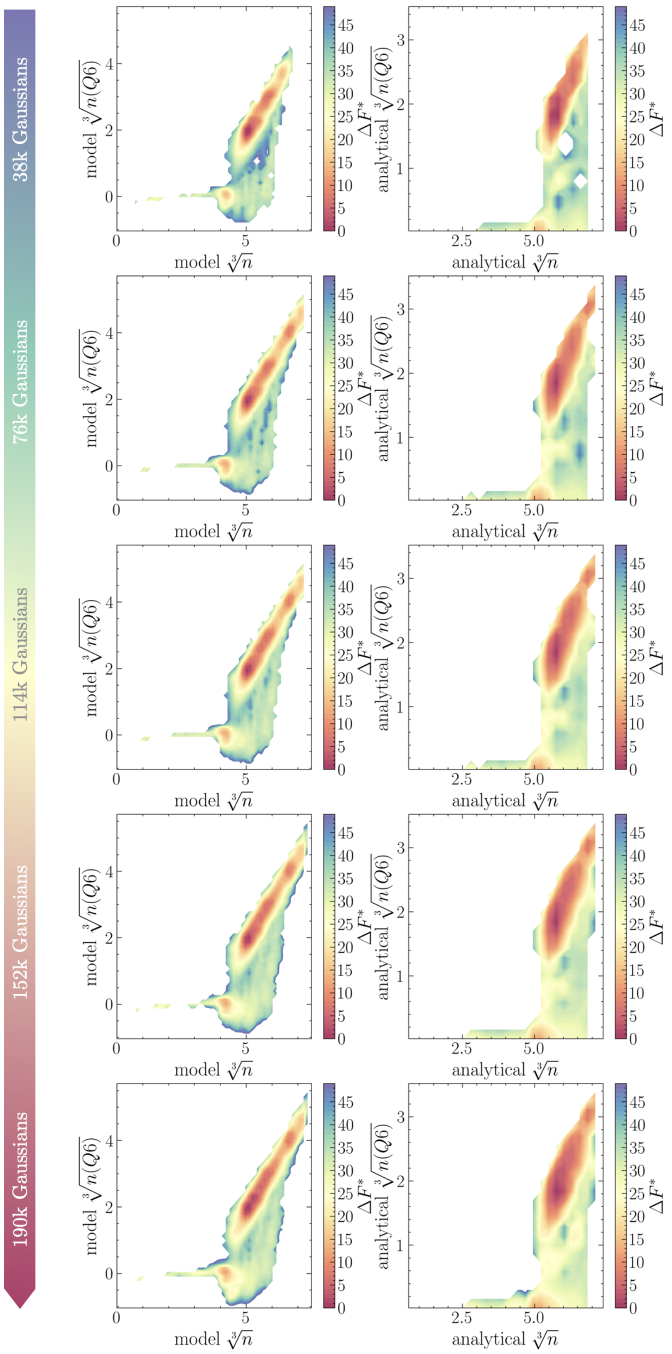

Figure S2: Convergent behaviour of the reweighted FES with an increasing number of samples from four independent metadynamics simulations. The *left* column shows the FES in the space of the *model* CVs. The *right* column displays the same result for the *analytical CVs*.

Merging Independent Metadynamics Simulations with MFI

Eq. S2 can be extended to naturally include the contribution from M multiple independent simulations as shown in Marinova and Salvalaglio [1]. The mean force can be obtained as a weighted average of the contribution of different simulations following the approach proposed by Umbrella Integration. [2] In such case the weight of the kth simulation, of length T_k steps, is defined as:

$$W_k = \sum_{t=1}^{T_k} p_t^b(\mathbf{s}) \tag{S5}$$

The combined mean force obtained from M independent simulations is thus:

$$\left\langle \nabla F_t(\mathbf{s}) \right\rangle_M = \frac{\sum_{k=1}^{M} W_k \left\langle \nabla F_{T_k}(\mathbf{s}) \right\rangle}{\sum_{k=1}^{M} W_k} \tag{S6}$$

Combining the mean force obtained from multiple metadynamics simulations enables the progressive refinement of the local mean force estimate across $\mathbf{s}$, and therefore leads to the estimate of a FES that accounts for the contribution from all simulations without the need to arbitrarily aligning FES obtained from independent runs. This approach can be extended to reweighted FES by defining the weight of a configuration obtained at time t, from the kth simulation is computed as:

$$w_{t,k} = T_k e^{\beta[V_{k,t}(\mathbf{s}) - c_k(t)]} \tag{S7}$$

where $V_{k,t}(\mathbf{s})$ is the total bias potential acting on $\mathbf{s}$ in simulation k at time t, T_k is the total time of simulation k, and $c_k(t)$ is the time-dependent constant $c(t)$ (Eq. S4) computed for simulation k. In Figure S2, we report the convergent behaviour of the reweighted FES obtained by combining an increasingly large number of frames sampled from the four independent simulations, reported in Figure S1, Table S1. In the left column, we show the combined FES obtained by reweighting the samples in the space of the *model* variables. In the right column, the same result is shown for the *analytical* variables. The FES obtained by combining all samples from the four independent metadynamics simulations is reported in Figure 7 of the main manuscript.

References

(1) Marinova, V.; Salvalaglio, M. Time-independent free energies from metadynamics via mean force integration. *The Journal of Chemical Physics* **2019**, *151*, 164115.

(2) Kästner, J.; Thiel, W. Bridging the gap between thermodynamic integration and umbrella sampling provides a novel analysis method: "Umbrella integration". *The Journal of chemical physics* **2005**, *123*.

(3) Zwanzig, R. W. High-temperature equation of state by a perturbation method. I. Nonpolar gases. *The Journal of Chemical Physics* **1954**, *22*, 1420–1426.

(4) Torrie, G.; Valleau, J. Nonphysical sampling distributions in Monte Carlo free-energy estimation: Umbrella sampling. *Journal of Computational Physics* **1977**, *23*, 187 – 199.

(5) Tiwary, P.; Parrinello, M. A time-independent free energy estimator for metadynamics. *The Journal of Physical Chemistry B* **2015**, *119*, 736–742.

(6) Bonomi, M.; Barducci, A.; Parrinello, M. Reconstructing the equilibrium Boltzmann distribution from well-tempered metadynamics. *Journal of computational chemistry* **2009**, *30*, 1615–1621.

(7) Tribello, G. A.; Bonomi, M.; Branduardi, D.; Camilloni, C.; Bussi, G. PLUMED 2: New feathers for an old bird. *Computer Physics Communications* **2014**, *185*, 604–613.

(8) Bonomi, M.; Bussi, G.; Camilloni, C.; Tribello, G. A.; Banáš, P.; Barducci, A.; Bernetti, M.; Bolhuis, P. G.; Bottaro, S.; Branduardi, D.; et al., Promoting transparency and reproducibility in enhanced molecular simulations. *Nature methods* **2019**, *16*, 670–673.
